# Impact of Techno-Creators and Techno-Inhibitors on Techno-Stress Manifestations in Chilean Kindergarten Directors in the Context of the COVID-19 Pandemic and Teleworking

**DOI:** 10.3389/fpsyg.2022.865784

**Published:** 2022-05-31

**Authors:** Carla Estrada-Muñoz, Alejandro Vega-Muñoz, Joan Boada-Grau, Dante Castillo, Sheyla Müller-Pérez, Nicolas Contreras-Barraza

**Affiliations:** ^1^Departamento de Ergonomía, Universidad de Concepción, Concepción, Chile; ^2^Departamento de Psicología, Universidad Rovira i Virgili, Tarragona, Spain; ^3^Public Policy Observatory, Universidad Autónoma de Chile, Santiago, Chile; ^4^Centro de Estudios e Investigación Enzo Faletto, Universidad de Santiago de Chile, Santiago, Chile; ^5^Facultad de Economía y Negocios, Universidad San Sebastián, Santiago, Chile; ^6^Facultad de Economía y Negocios, Universidad Andres Bello, Viña del Mar, Chile

**Keywords:** mental health, technostress, techno-creators, techno-inhibitors, information-technology, education, work, information overload

## Abstract

The research objective was to predict the impact of techno-creators and techno-inhibitors on the different manifestations of technostress in kindergarten directors in the context of the COVID-19 pandemic and telework. The participants were INTEGRA Foundation kindergarten directors, from a sample of 567 kindergartens in Chile. To measure the technostress manifestations, the RED-TIC questionnaire was used as an instrument, and concerning techno-creators and techno-inhibitors, those established in previous research were considered. The partial least squares structural equation modeling (PLS-SEM) methodology was used, and the model estimation was performed using SmartPLS version 3.0 software. It was obtained that techno-creators correlate positively and significantly with the technostress manifestations. A negative correlation was found between techno-inhibitors and technostress manifestations and techno-creators, but not significant for skepticism and inefficacy manifestations. Therefore, it is concluded that techno-creators lead to technostress manifestations, however, techno-inhibitors did not show a significant effect in reducing these manifestations in the sample studied.

## Introduction

The incorporation of information and communication technologies (ICTs) into people's daily lives has had both positive and negative side effects (MacKay and Vogt, 2012). Although ICTs offer indisputable benefits, such as allowing to maintain family contact, especially in remote or rural locations, which would have a positive impact on people's wellbeing, or others, such as facilitating work allowing to take advantage of waiting times and adapting work times to the working people's needs (Pearson et al., 2017). A special attention should be paid to the possible negative consequences, since their use at work can generate technostress given the mental demands required by their use (Macías-García, 2019).

Technostress is defined according to Tarafdar et al. (2007) as the stress experienced by people due to the use of information systems, derived from the demands that these systems cause on the individual and its study has been increasing exponentially in recent decades (Bondanini et al., 2020; Salazar-Concha et al., 2021). Among the consequences of occupational technostress are decreased job satisfaction, decreased user commitment to the organization, increased conflict, and role overload, reduced productivity, performance, and innovation during their use at work (Tarafdar et al., 2007, 2010, 2011, 2014; Jena, 2015).

Regarding wellbeing, the World Health Organization (World Health Organization, 2021) incorporates this concept in the health definition, describing it as a state of complete physical, mental, and social wellbeing, and not only the absence of disease or illness. The International Labour Organization (1995) also mentions this concept within the occupational health objective, which defines it as the promotion and maintenance of the highest degree of physical, mental, and social wellbeing in workers in all occupations. On the other hand, Martínez (2011) incorporates the wellbeing notion when defining technostress, referring to it as a manifestation that hurts the physical and mental wellbeing of the almost mandatory ICT implementation in the work, leisure, and private life spheres.

But in a more specific and relevant to this study, Diener et al. (2002) define subjective wellbeing as “cognitive and affective evaluations of one's own life; these evaluations include emotional reactions to events, as well as cognitive satisfaction judgments. Thus, subjective wellbeing is a broad concept that includes pleasant emotions, low negative mood levels, and high life satisfaction” (p. 63), and subjective wellbeing can also be defined as a construct composed of a cognitive component that alludes to people's satisfaction and their satisfaction with specific or global aspects of their existence, and an affective component, which refers to positive mood states (García-Viniegras and González, 2000; Arita, 2005). Moreover, Diener (2006) adds that subjective wellbeing consists of the different evaluations that people do of their lives, the events that take place in them, their bodies and minds, and the circumstances in which they live.

The environment in which activities are developed, the work environment, or other factors derived from working conditions, associated with the life rhythm that is imposed nowadays, can lead to occupational stress in workers (Macías-García, 2019). Thus, conditions that create technostress can be considered stressors and constitute work demands that require effort on the part of workers leading to tension and stress feelings (Pfaffinger et al., 2020), and negative work outcomes (Srivastava et al., 2015), which lead to a reduction in workers' wellbeing (Paschoal et al., 2015).

Therefore, technostress should be considered a particular threat to wellbeing (Nimrod, 2018). Also, the lack of wellbeing in teachers affects the academic performance of students, and in the case of teachers, it can produce bewilderment, dissatisfaction, transfer requests, desires to leave school, absenteeism, burnout, stress, feelings of guilt, reactive neuroses, depressions, anxiety, etc. (Hué, 2009). To promote wellbeing, the work environment must be adapted to the needs of workers (Stich et al., 2019). According to Molino et al. (2020a), technology use acceptance has been positively associated with work engagement, which is related to workers' sense of wellbeing. On the other hand, wellbeing is found to be related to job satisfaction (Barrientos, 2005) and good job performance (Pavot and Diener, 2004).

The COVID-19 pandemic, which started on December 1, 2019, in Wuhan City, China (Huang et al., 2020), made most organizations face the challenge of introducing telework practices, because of the health measures proposed by the health authorities (Angelici and Profeta, 2020; Tokarchuk et al., 2021). Telework refers to the performance of work generated with the ICT support and performed outside the established organization (Belzunegui-Eraso and Erro-Garcés, 2020).

Due to the emergency installation of this telework modality using information and communication technologies during the pandemic of COVID-19, several studies have been conducted on technostress at different educational levels, for example, at the level of primary and secondary education, higher stress levels have been reported in teachers due to online education (Truzoli et al., 2021), with greater anxiety and fatigue manifestations for female teachers (Estrada-Muñoz et al., 2021), and decreased job performance (Cahapay and Bangoc, 2021). On the other hand, at the higher education level, in the study by Dahabiyeh et al. (2022), it is mentioned that the technostress creators were associated with burnout and decreased teacher productivity, and in the research by Penado-Abilleira et al. (2021), it was found that the teachers who suffered the most from the negative technological consequences were those who were older, with more years of experience and, consequently, who held a higher position.

Although the adoption of teleworking allows the operation of educational institutions to continue and maintain contact between work teams (Ramadani et al., 2020), the conditions related to the use of technologies can be creators of technostress, affecting the wellbeing of workers, the establishment of mitigation measures that inhibit technostress becoming relevant (Jena, 2015), is that the objective of this research is to predict the impact of techno-creators and techno-inhibitors on the different manifestations of technostress in directors of kindergartens. In the context of the COVID-19 pandemic and teleworking.

## Literature Review

### Technostress Manifestations

According to Salanova (2003), technostress is a negative psychological state related to the use of ICTs resulting from the perception of a mismatch between technological demands and the personal resources available to face the use of these technologies, which leads to a high unpleasant psychophysiological activation level, and to the development of negative attitudes and thoughts toward the technology use and the individual capacity to use them. In this sense, if technostress manifests itself with a high unpleasant physiological activation level, we speak of techno anxiety, and if it does so with tiredness and exhaustion feelings, we speak of techno fatigue; in both cases accompanied by skeptical attitudes and ineffectiveness beliefs (Salanova et al., 2007). Therefore, the variables that construct technostress correspond to anxiety, fatigue, skepticism, and ineffectiveness produced by the interaction with technology (Salanova et al., 2007).

### Precursors of Technostress

There are mainly five precursor conditions of technostress, described as techno-creators, to which ICT users may be subjected; these conditions correspond to techno-overload, techno-invasion, techno-complexity, techno-insecurity, and techno-uncertainty (Tarafdar et al., 2011; Jena, 2015). Techno-overload refers to the need for information processing of different tasks simultaneously with the use of technological devices; techno-invasion occurs when technology invades personal life and privacy, with the need to be constantly connected anywhere and at any time; techno-complexity is defined as the complexity associated with the use of ICTs that makes it necessary to spend time and effort learning how to use them; techno-insecurity is the feeling that technology threatens the maintenance of employment; and techno-uncertainty is a stress factor due to the constant updates and changes in ICTs, which do not allow users to develop an experience base (Tarafdar et al., 2007, 2011; Jena, 2015). Given the above, this research examines the influence of techno-creators on the manifestations of technostress.

**Figure 1:**
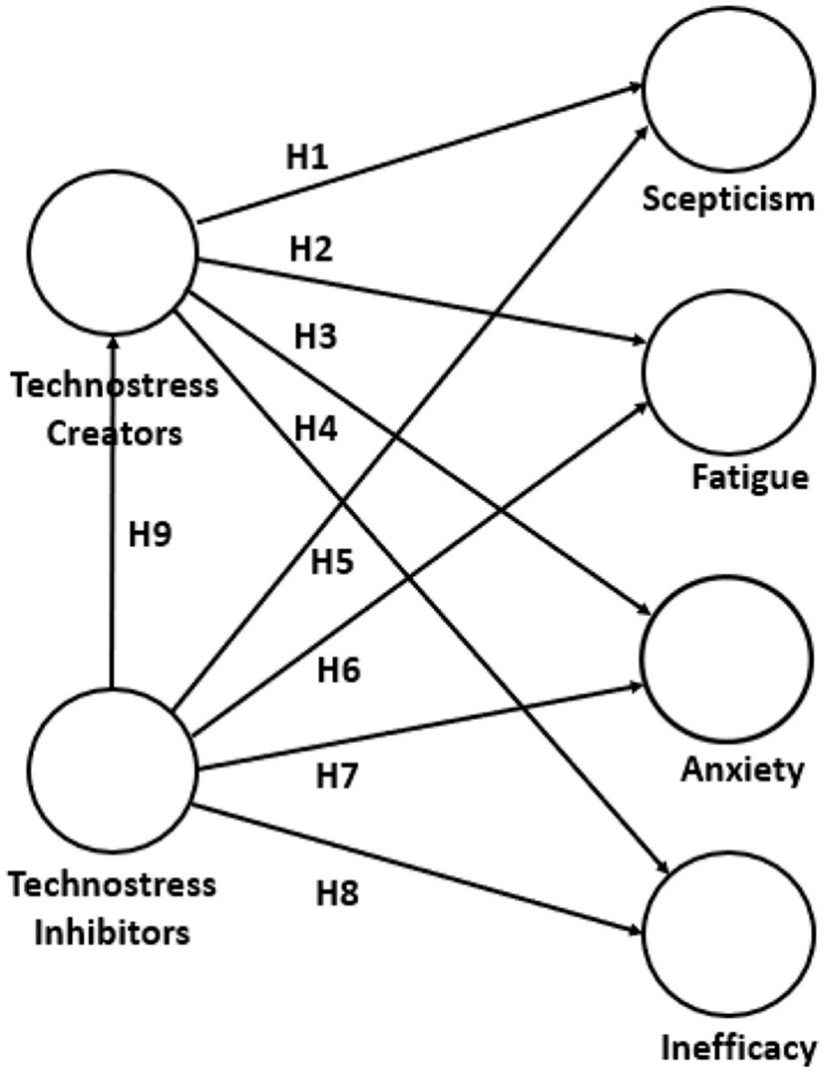
The conceptual model under study.

Then, techno-creators, leading to technostress manifestations, have affectations at different levels, both personal and occupational; at the personal level, techno-creators can affect health (Ayyagari et al., 2011; Jena, 2015), provoke negative emotions (Wang et al., 2020), generate prolonged stress (Salo et al., 2019), and even induce work-family conflict (Molino et al., 2020b). At the workplace level, techno-creators have been associated with decreased job satisfaction (Al-Ansari and Alshare, 2019), organizational commitment (Hung et al., 2015), job performance (Christ-Brendemühl and Schaarschmidt, 2020), and productivity (Tiwari, 2021). In this research, the following techno-creators reported by Jena (2015) are considered: to be forced by ICT to live with very tight time schedules, to be forced to change habits to adapt to new developments in technology, to have to sacrifice the personal time to keep current on latest technologies, feel that the personal life is being invaded by ICT and not to find enough time to study and upgrade the technical skills. The following hypotheses are presented in this regard (see Figure 1).

H1: Techno-creators correlate positively with skepticism.
H2: Techno-creators correlate positively with fatigue.
H3: Techno-creators correlate positively with anxiety.
H4: Techno-creators correlate positively with inefficacy.

### Technostress-Inhibitors

There are situational conditions, called technostress-inhibitors, involved in the stress reduction derived from the use of ICT, which would act as moderators, playing an important role in reducing the non-beneficial consequences caused by the introduction of these technologies in organizations (Ragu-Nathan et al., 2008; Tarafdar et al., 2019). Techno-inhibitors, when implemented in organizations, could mitigate the stress associated with ICT use by decreasing the techno-creator effects resulting from technology demands (Salanova et al., 2007; Fuglseth and Sørebø, 2014). The main ones reported in the literature are, facilitating literacy, which allows increasing technological knowledge; organizational technical support; facilitating participation, involving workers in the adoption and development of ICT; and supporting innovation, through mechanisms that encourage experimentation and learning (Tarafdar et al., 2011; Li and Wang, 2021). Moreover, in this research, the influence of technostress inhibitors on the technostress manifestations and techno-creators is examined.

In this research, the following techno-inhibitors reported by Jena (2015) are considered: the organization provides clear documentation to use new technologies, the organization emphasizes teamwork in dealing with new technology-related problems, the technology help desk is responsive to end-user requests, the organization rewards for using new technologies, and the organization consults before the introduction of new technology. The hypotheses put forward are mentioned below (see Figure 1):

H5: Techno-inhibitors will correlate negatively with skepticism.
H6: Techno-inhibitors will correlate negatively with fatigue.
H7: Techno-inhibitors correlate negatively with anxiety.
H8: Techno-inhibitors correlate negatively with inefficacy.
H9: Techno-inhibitors correlate negatively with techno-creators.

Thus, the conceptual model in Figure proposes to test whether techno-creators are positively correlated with technostress manifestations and whether techno-inhibitors are negatively correlated with technostress manifestations and techno-creators.

## Materials and Methods

### Participants

The kindergarten directors of Fundación INTEGRA participated, based on a sample of 567 kindergartens in Chile.

#### Respondent Characterization

Table 1 shows the characteristics of the respondents, who are distributed regionally according to Chilean population concentrations and the presence of Fundación INTEGRA's educational centers. A high percentage of these kindergartens are in urban areas (81.7%), serving groups of <190 infants and children (94%), and whose directors have between 1 and 38 years of work experience in education, with a mean of 16.4 years (σ = 7.3 years), who are identified in 99.5% of the cases as female, and who are identified in 99.5% of the cases as being of the female gender. They have between 0 and 100 collaborators in charge with an average of 19 people (σ = 13 persons), their age fluctuates between 24 and 65 years ($\bar{x}$ = 42; σ = 8 years), and they live with between 0 and 14 people ($\bar{x}$ = 3; σ = 3 persons).

**Table 1:** Respondent characteristics.

	**Frequency**	**Percent**
**Region**		
Santiago Metropolitan	118	20.8%
Maule	59	10.4%
Los Lagos	56	9.9%
La Araucanía	51	9.0%
Biobío	50	8.8%
Valparaíso	49	8.6%
Libertador General Bernardo O'Higgins	44	7.8%
Coquimbo	32	5.6%
Ñuble	24	4.2%
Los Ríos	22	3.9%
Tarapacá	18	3.2%
Aysén of General Carlos Ibáñez del Campo	12	2.1%
Antofagasta	11	1.9%
Atacama	8	1.4%
Magallanes and the Chilean Antarctica	7	1.2%
Arica and Parinacota	6	1.1%
Total	567	100.0%
**Area (urbanization)**		
Urban	463	81.7%
Rural	104	18.3%
Total	567	100.0%
**Kindergarten category**		
A (190 ≤ babies and children)	34	6.0%
B (100 ≤ babies and children <190)	166	29.3%
C (babies and children <100)	367	64.7%
Total	567	100.0%
**Working-age (years, y)**		
0 < y ≤ 10	130	22.9%
10 < y ≤ 20	286	50.4%
20 < y ≤ 30	138	24.3%
30 < y	13	2.3%
Total	567	100.0%
**Team of collaborators (c)**		
0	16	2.8%
0 < c ≤ 10	139	24.5%
10 < c ≤ 20	175	30.9%
20 < c ≤ 30	146	25.7%
30 < c	91	16.0%
Total	567	100.0%
**Gender**		
Female	564	99.5%
I prefer not to say it	3	0.5%
Total	567	100.0%
**Age (years old, y)**		
20 < y ≤ 30	32	5.6%
30 < y ≤ 40	226	39.9%
40 < y ≤ 50	224	39.5%
50 < y ≤ 60	78	13.8%
60 < y	7	1.2%
Total	567	100.0%
**Number of people you live with**		
0	22	3.9%
1	52	9.2%
2	121	21.3%
3	172	30.3%
4	120	21.2%
5 or more	80	14.1%
Total	567	100.0%

### Procedure

A national self-response survey was applied to a sample of directors of kindergartens and/or nurseries in the Integra Foundation in the context of the COVID-19 pandemic, and telework during the second semester of 2021, which considered the measurement of the technostress manifestations, techno-creators and techno-inhibitors, and a respondent's sociodemographic characterization.

To measure the manifestations of technostress, the RED-TIC questionnaire is used as an instrument (Salanova et al., 2007), previously employed in the Chilean educational setting (Estrada-Muñoz et al., 2020, 2021; Vega-Muñoz and Estrada-Muñoz, 2020), and composed of 16 items, which are queried using a Likert – type scale (never – 0, a couple of times a year – 1, once a month – 2, a couple of times a month – 3, once a week – 4, a couple of times a week – 5, every day – 6).

Regarding techno-creators and techno-inhibitors, we consider those established in Jena's (2015) research, composed of 5 items each, which were measured on a Likert-type scale (strongly disagree – 0, disagree – 1, neither agree nor disagree – 2, agree – 3, and strongly agree – 4).

On the other hand, a respondents characterization was made, considering the following: region in Chile from where they were performing their work in telework mode, urban or rural kindergarten/daycare center sector, kindergarten/daycare category center according to the children attending number, years working in the educational field, dependents number, gender, age and the number of people with whom they live.

### Statistical Analysis

Since the article's objective is to predict the impact of technostress-creators and technostress-inhibitors on the different technostress manifestations, the partial least squares structural equation modeling (PLS-SEM) is the methodology that allows for meeting this objective, given its predictive power, (Hair et al., 2017; Weidlich and Bastiaens, 2017; García-Fernández et al., 2018; Shmueli et al., 2019; Tan et al., 2020; Zhang et al., 2020; Dash and Paul, 2021; Al-Jundi et al., 2022).

According to Hair et al. (2017), PLS-SEM models allow the prediction of key constructs, and their evaluation comprises 2 stages: the measurement models evaluation and the structural model evaluation. Using empirical measures, the first stage seeks to determine the relationships between the items and constructs, and the second stage focuses on the relationship between the constructs of the theoretical model established.

#### First Phase: Measurement Model Evaluation

The proposed theoretical model is only composed of reflective measures, i.e., the items are manifestations of the established constructs. Therefore, the reflective measurement model must show that the items that make up the construct have internal consistency (Cronbach's alpha and Composite Reliability) and Convergent Validity, which allows identifying how the items belonging to a construct correlate (Outer Loading, Average Variance Extracted). Finally, it is necessary to ensure that the constructs comply with the Discriminant Validity, which is to ensure that a construct is unique and different from the other constructs of the established model (no interval of the construct combinations the Heterotrait-Monotrait Ratio (HTMT) must include the value of 1).

#### Second Phase: Structural Model Evaluation

Once it is verified that the constructs are reliable and discriminant, the model is validated. First, the constructs' collinearity that makes up the model is ruled out, then the bootstrapping technique that is used to check that the hypothesis's directions (signs) are those established in the theoretical model, and the relationships relevance of each hypothesis is examined to ensure that they are significant. To determine the model predictive power within the sample, the adjusted *R*^2^ coefficient of determination is used, but to check the model predictive relevance outside the sample, the blindfolding technique is used, which gives the Stone-Geisser *Q*^2^ value. Finally, the effect on the predictive power (*f*^2^) and relevance (*q*^2^) of each endogenous construct when each construct is omitted from the model is evaluated.

Table 2 presents the criteria to be met by each of the model evaluation phases. The PLS-SEM model estimation was performed using SmartPLS version 3.0 software (Ringle et al., 2015).

**Table 2:** Evaluation criteria.

Evaluation of the reflective measurement model	Internal consistency reliability	Cronbach's alpha (α) ≥ 0.70
		Composite Reliability (CR) ≥ 0.70
	Convergent Validity	Outer loading ≥ 0.70
		Average Variance Extracted (AVE) ≥ 0.50
	Discriminant validity	Confidence interval HTMT doesn't have 1
Evaluation of the structural model	Collinearity: Variance Inflation Factor (VIF) < 5	
	Predictive power (*R*^2^ adjusted): 0.25 (weak), 0.50 (moderate) and 0.75 (significant).	
	Magnitude and significance of the path coefficients when *p*-value ≤ 0.05.	
	Predictive relevance *Q*^2^ >0	
	Effect size (*f*2): values of 0.02, 0.15 and 0.35 are considered small, moderate, and large effects.	
	Effect size (*q*2): values of 0.02, 0.15 and 0.35 are considered small, moderate, and large effects.	

## Results

### Reflective Measurement Model Evaluation

Tables 3, 4 show that the model constructs meet the criteria for convergent and discriminant validation. Regarding convergent validation 2 items, r15 and t9, present external loadings below 0.7, but the recommendation of Hair et al. (2017) to keep these items in the construct is followed, since their Average Variance Extracted (AVE) is >0.50.

**Table 3:** Results of reflective measurement model evaluation.

**Factor**	**Item**	**Convergent validity**		**Internal consistency reliability**		**Discriminant validity**
		**Outer loading**	**AVE ≥ 0.50**	**Cronbach's**	**Composite**	**Confidence interval**
		**≥ 0.70**		**α ≥ 0.70**	**Reliability (CR) ≥ 0.70**	**HTMT doesn't have 1**
Skepticism	r_1	0.802	0.808	0.796	0.866	Yes
	r_2	0.738				
	r_3	0.804				
	r_4	0.801				
Fatigue	r_5	0.859	0.922	0.918	0.942	Yes
	r_6	0.911				
	r_7	0.917				
	r_8	0.898				
Anxiety	r_9	0.849	0.893	0.874	0.913	Yes
	r_10	0.827				
	r_11	0.865				
	r_12	0.859				
Inefficacy	r_13	0.809	0.908	0.836	0.878	Yes
	r_14	0.871				
	r_15	0.592^*^				
	r_16	0.726				
Technostress creators	t_1	0.762	0.861	0.857	0.898	Yes
	t_2	0.808				
	t_3	0.841				
	t_4	0.833				
	t_5	0.745				
Technostress inhibitors	t_6	0.797	0.833	0.815	0.868	Yes
	t_7	0.784				
	t_8	0.753				
	t_9	0.684^*^				
	t_10	0.751				

* *External loads below 0.7*.

**Table 4:** Confidence Intervals.

**Interval**	**2.50%**	**97.50%**
SKE -> ANX	0.533	0.684
FAT -> ANX	0.682	0.778
FAT -> SKE	0.485	0.632
INE -> ANX	0.916	0.986
INE -> SKE	0.547	0.703
INE -> FAT	0.653	0.742
TC -> ANX	0.503	0.633
TC -> SKE	0.273	0.436
TC -> FAT	0.617	0.715
TC -> INE	0.432	0.562
TI -> ANX	0.126	0.273
TI -> SKE	0.093	0.200
TI -> FAT	0.162	0.320
TI-> INE	0.116	0.206
TI -> TC	0.152	0.318

### Evaluation of the Structural Model

The model does not present critical collinearity levels as shown in Table 5, so it is possible to estimate the power and predictive relevance of the model.

**Table 5:** Inner Variance Inflation Factor (VIF) values.

**Factors**	**ANX**	**FAT**	**INE**	**SKE**	**TC**
TC	1.042	1.042	1.042	1.042	
TI	1.042	1.042	1.042	1.042	1.000

**Figure 2:**
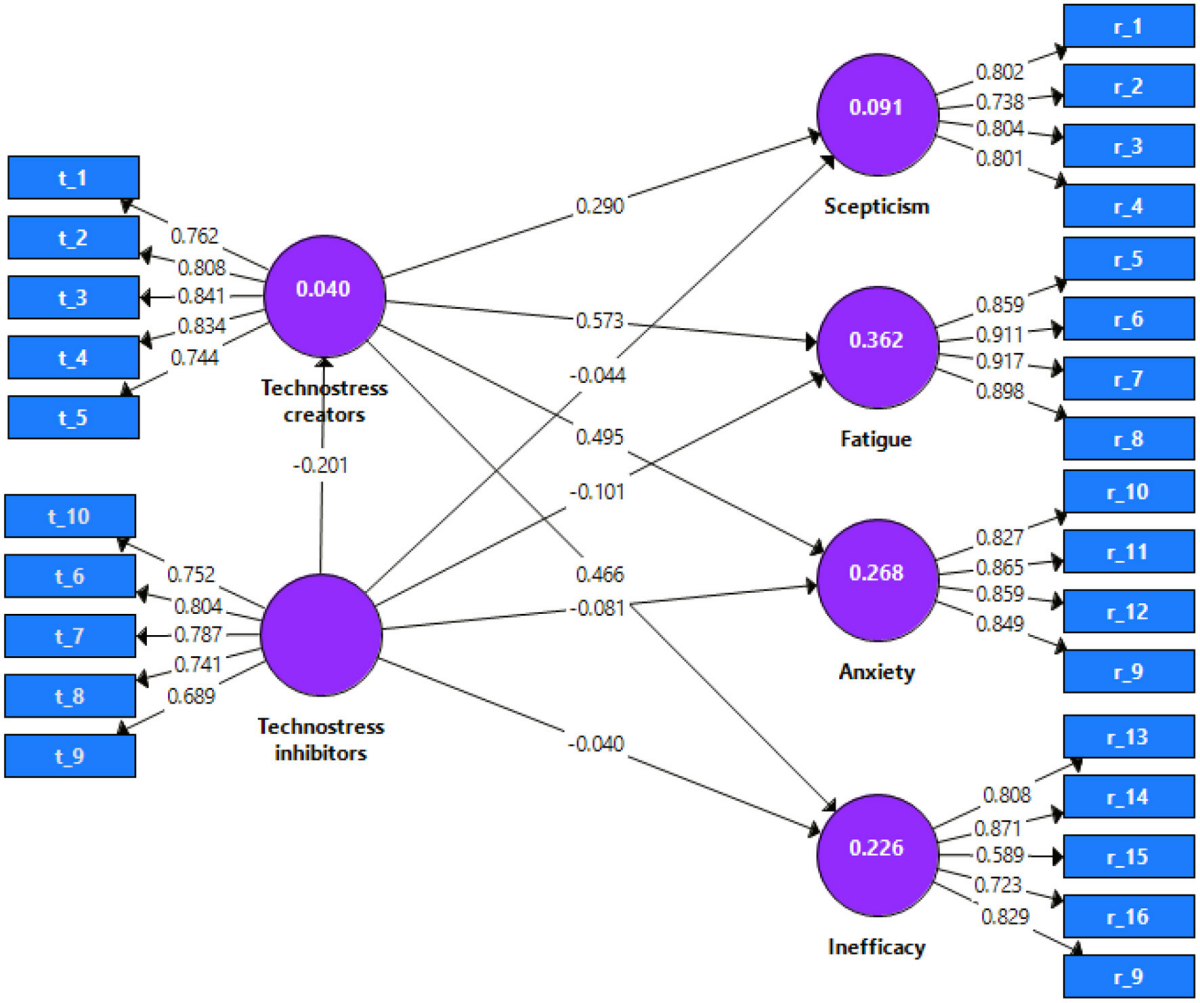
Structural model evaluation results.

Figure 2 shows the final estimates of the model and with the data in Table 6, it can be affirmed that only hypotheses 5 and 8 are not significant. It can also be seen that Technostress Creators (TC) have a positive and strong relationship with the fatigue factor (FAT, 0.573), as well as a positive and moderate relationship with the factors Anxiety (ANX, 0.495), Inefficacy (INE, 0.466), and Skepticism (SKE, 0.290). Regarding Technostress Inhibitors, the model shows a weak negative relationship with the factors Technostress Creators (TC, −0.201), Fatigue (FAT, −0.101), and Anxiety (ANX, −0.081).

**Table 6:** Structural model evaluation results.

**Hypothesis**	**Path**	**Path coefficients**	***P*-value**	**Result**
H1	TC -> ANX	0.495	0.000	Yes
H2	TC -> SKE	0.290	0.000	Yes
H3	TC -> FAT	0.573	0.000	Yes
H4	TC -> INE	0.466	0.000	Yes
H5	TI -> SKE	−0.044	0.134	No
H6	TI -> FAT	−0.101	0.003	Yes
H7	TI -> ANX	−0.081	0.025	Yes
H8	TI -> INE	−0.040	0.153	No
H9	TI -> TC	−0.201	0.000	Yes

Regarding predictive power and relevance in Table 7, the Anxiety (ANX) and Fatigue (FAT) factors have moderate predictive power (0.360, 0.265, respectively). On the other hand, the Inefficacy (INE), Skepticism (SKE), and Technostress Creators (TC) factors have a weak predictive power (0.222, 0.088, and 0.039, respectively). All Q^2^ values being greater than zero, the factors have predictive relevance.

**Table 7:** Power and predictive relevance results.

**Factor**	***R* ^2^**	***Q* ^2^**	***f* ^2^**	***f* ^2^**	***q* ^2^**	***q* ^2^**
	**Adjusted**		**exclude**	**exclude**	**exclude**	**exclude**
			**TC**	**TI**	**TC**	**TI**
ANX	0.265	0.180	0.321	0.009	0.196	0.002
SKE	0.088	0.051	0.088	0.002	0.048	0.000
FAT	0.360	0.285	0.494	0.015	0.350	0.008
INE	0.222	0.114	0.269	0.002	0.121	−0.001
TC	0.039	0.025		0.042		

The impact on the predictive power (*f*^2^) of excluding Technostress creators from the model is large for the fatigue factor (0.494), the effect is moderate for the anxiety (0.321), and inefficacy (0.269) factors, and there is a weak impact on the skepticism factor (0.088). On the other hand, excluding Technostress inhibitors does not show large or moderate effects, but small effects, so this construct could be eliminated from the model. The effect on the predictive significance (*q*^2^) of excluding Technostress creators from the model is large for the fatigue factor (0.350), the effect is moderate for the anxiety factor (0.196), and there is a weak impact on the inefficacy (0.121) and skepticism (0.048) factors. On the other hand, when excluding Technostress inhibitors, small effects are presented in the model predictive relevance, which confirms that this construct does not contribute to predicting the factors of manifestation of technostress.

## Discussion

This research aimed to predict the impact of techno-creators and techno-inhibitors on the different technostress manifestations in kindergarten directors in the context of the COVID-19 pandemic and telework. The manifestations described by Salanova et al. (2007) were considered, and as for the techno-creators and techno-inhibitors, those established in the research by Jena (2015) were included.

According to the research results, it is verified that techno-creators (Jena, 2015) correlate positively and significantly with the technostress manifestations, as described by Salanova et al. (2007). In this sense, it is worth noting that, this positive correlation is strong for fatigue, and moderate for skepticism, anxiety, and inefficacy. In other words, the techno-creators considered lead to technostress manifestations in the sample studied. These positive correlations coincide with previous research, which also mentions that the main techno-creators leading to technostress correspond to techno-overload, techno-invasion, and techno-insecurity (Ayyagari et al., 2011; Suh and Lee, 2017; Florkowski, 2019).

On the other hand, regarding the correlation between techno-inhibitors (Jena, 2015) and technostress manifestations (Salanova et al., 2007), it was found that, although the correlations are negative, which supports the hypotheses raised, the correlation between techno-inhibitors and the skepticism and ineffectiveness manifestations, is not significant, and as for the fatigue and anxiety manifestations, a weak negative correlation is shown, as well as when correlating techno-inhibitors with techno-creators. Even though studies such as Califf and Brooks (2020), where it is argued that literacy facilitation acts as a techno-inhibitor on techno-creators such as techno-complexity, techno-insecurity, techno-invasion, and techno-overload, Hang et al. (2022), in which it is mentioned that techno-inhibitors such as literacy facilitation and the provision of technical support help workers cope with technostress, neutralizing the negative effects of techno-creators, the evidence to reliably support that the most commonly reported techno-inhibitors in the literature have a relevant impact on the technostress manifestations and techno-creators, is scarce, and even contradictory results are reported. In this regard, Jena (2015) is cited, who argues that techno-inhibitors restrain techno-creators, however, according to Li and Wang (2021), literacy facilitation programs, as a techno-inhibitor, could stimulate the development of techno-creators, as they may add new sources of stress.

Most studies argue that techno-stressors are associated with turnover intention (Califf and Brooks, 2020), adverse work outcomes (Borle et al., 2021), and significantly and negatively affect workers' well-being (Salo et al., 2019; Hang et al., 2022). According to the research of González-López et al. (2021), technostress, at the individual level is related to, abandonment of daily activities, increased loneliness, lack of concentration, irregular sleep patterns, avoidance of real-life problems, reduced hygiene and eating problems, at the group level with, social, family and privacy problems, and at the professional level with, absenteeism, missed deadlines and failure to achieve objectives.

Studies in the context of the COVID-19 pandemic highlight that telework is associated with technostress (Hinojosa-López et al., 2021) and work-home conflict, decreasing job satisfaction and performance (Camacho and Barrios, 2022). Therefore, it is important to inquire about what measures are most effective to inhibit technostress at work, especially in the educational system (Chauhan, 2017), where the use of ICT in telework mode, during the pandemic of COVID-19 became imperative (Sangster et al., 2020).

Regarding the effect of techno-inhibitors on manifestations of technostress, research must be extended to other factors proposed in the literature, in addition to the classic factors, such as, for example, cultural segmentation, which refers to the organizational culture that favors the separation between work and personal life (Kim et al., 2015), the establishment of breaks during the working day (Tarafdar et al., 2019), or other strategies such as the implementation of positive technology, scientific and applied approach to the use of technology to improve the quality of personal experience that can lead to increase the wellbeing of workers and prevent technostress (Brivio et al., 2018).

From a practical point of view, imposing the use of ICTs without considering the capabilities, needs, and limitations of workers, and without implementing strategies to mitigate the risks associated with the use of these technologies, can generate technostress. Thus, this research contributes to increasing knowledge regarding the influence of techno-creators and techno-inhibitors on the technostress manifestations, making available to practitioners which are the factors that most affect technostress, and based on this, generating strategies to prevent the conditions that contribute to increasing the stress associated with the use of ICT in the workplace, to provide and promote healthy work environments that promote wellbeing in workers.

## Conclusion

Based on the research results, it is concluded that techno-creators and techno-inhibitors correlate positively and negatively, respectively, with manifestations of anxiety, skepticism, fatigue, and ineffectiveness, and that techno-inhibitors have a negative association with techno-creators, in the kindergarten directors who participated in the study in the COVID-19 pandemic and telework context. Specifically for the techno-creators case, all correlations were significant, which allows corroborating their impact and prediction on the technostress manifestations, and for the techno-inhibitors case, it is not predictable their influence on techno-creators and the technostress manifestations, especially for skepticism and ineffectiveness since the correlations were not significant. Therefore, the techno-inhibitors considered in the studied sample did not show the expected effect, which is to generate a significant reduction in the technostress manifestations.

## Data Availability Statement

The original contributions presented in the study are included in the article/Supplementary Material, further inquiries can be directed to the corresponding author/s.

## Author Contributions

AV-M and CE-M: conceptualization, formal analysis, and project administration. DC and SM-P: methodology. SM-P: software. AV-M and JB-G: validation. DC, AV-M, and SM-P: data curation. CE-M, NC-B, and SM-P: writing—original draft preparation. AV-M: writing—review and editing. JB-G: supervision. AV-M, NC-B, and DC: funding acquisition for publishing fees. All authors have read and agreed to the published version of the manuscript.

## Conflict of Interest

The authors declare that the research was conducted in the absence of any commercial or financial relationships that could be construed as a potential conflict of interest.

## Publisher's Note

All claims expressed in this article are solely those of the authors and do not necessarily represent those of their affiliated organizations, or those of the publisher, the editors and the reviewers. Any product that may be evaluated in this article, or claim that may be made by its manufacturer, is not guaranteed or endorsed by the publisher.
